# Growth differentiation factor-15 as a biomarker of atherosclerotic coronary plaque: Value in people living with and without HIV

**DOI:** 10.3389/fcvm.2022.964650

**Published:** 2022-08-26

**Authors:** Léna Royston, Stéphane Isnard, Nils Perrin, Liliya Sinyavskaya, Carolina Berini, John Lin, Benoit Trottier, Jean-Guy Baril, Carl Chartrand-Lefebvre, Cecile Tremblay, Madeleine Durand, Jean-Pierre Routy

**Affiliations:** ^1^Infectious Diseases and Immunity in Global Health Program, Research Institute, McGill University Health Centre, Montreal, QC, Canada; ^2^Chronic Viral Illness Service, McGill University Health Centre, Montreal, QC, Canada; ^3^CIHR Canadian HIV Trials Network, Vancouver, BC, Canada; ^4^Division of Infectious Diseases, Geneva University Hospital, Geneva, Switzerland; ^5^Structural Heart Intervention Program, Montreal Heart Institute, Montreal, QC, Canada; ^6^Centre de Recherche du Centre Hospitalier de l'Université de Montréal (CRCHUM), Montréal, QC, Canada; ^7^Instituto de Investigaciones Biomédicas en Retrovirus y SIDA (INBIRS), CONICET - Universidad de Buenos Aires, Buenos Aires, Argentina

**Keywords:** GDF-15, atherosclerosis, HIV, inflammation, coronary plaque

## Abstract

**Background:**

Increased rates of cardiovascular diseases (CVD) and larger subclinical high-risk coronary plaques in coronary CT angiography have been observed in people living with HIV (PLWH) treated with antiretroviral therapy (ART) compared to HIV-uninfected people. Growth differentiation factor-15 (GDF-15) is a cytokine emerging as an optimal marker for CVD in the general population.

**Methods:**

We cross-sectionally analyzed plasma of 95 PLWH on ART and 52 controls. We measured GDF-15, fibroblast growth factor-21 (FGF-21), glucagon-like peptide-2 (GLP-2), soluble urokinase plasminogen activator receptor (suPAR), CRP, and anti-CMV and anti-EBV IgG levels. All participants had no clinical CVD and underwent coronary CT angiography with the 3D reconstruction of coronary artery atherosclerotic plaques. Total plaque volume (TPV) and low attenuation plaque volume (LAPV, defined as density <30 Hounsfield Units) were calculated (mm^3^).

**Results:**

In both PLWH and controls, GDF-15 levels were increased in participants with presence of coronary plaque vs. without (*p* = 0.04 and *p* < 0.001, respectively) and correlated with TPV (*r* = 0.27, *p* = 0.009 and *r* = 0.62, *p* < 0.001, respectively) and LAPV (*r* = 0.28, *p* = 0.008, *r* = 0.60, *p* < 0.001, respectively). However, in a multivariate model, GDF-15 was independently associated with LAPV in controls only (adjusted OR 35.1, *p* = 0.04) and not in PLWH, mainly due to confounding by smoking. Other markers were not independently associated with plaque volume, except for anti-EBV IgGs in controls (adjusted OR 3.51, *p* = 0.02).

**Conclusion:**

In PLWH, GDF-15 and smoking seemed to synergistically contribute to coronary plaque volume. Conversely, increased GDF-15 levels were associated with the presence of coronary artery plaques in people without HIV, independently of CV risk factors.

## Introduction

Although antiretroviral treatment (ART) has dramatically improved the lives of people living with HIV (PLWH), these treatments are not sufficient to eradicate HIV. Prevention and treatment of non-AIDS comorbidities have become a new challenge in the management of these patients. Notably, PLWH exhibits a higher risk of cardiovascular diseases (CVD), including myocardial infarction (MI), which occur at a younger age than in the general population (1). Recent cohort studies reported a 1.5- to 3-fold increased risk for experiencing a MI in PLWH on ART (2–6). In a population-based cohort study analyzing primary care data of 45,000 individuals in the UK, PLWH had 50% more risk of CVD (composite endpoint including MI, ischemic heart disease, peripheral vascular disease, and heart failure) than matched HIV-uninfected individuals (6). However, the mechanisms contributing to PLWH's increased susceptibility to CVD remain poorly understood. Part of this increased risk may be explained by a higher incidence of traditional cardiovascular risk, especially tobacco smoking. Smoking incidence has thus been estimated to be 2- to 3-fold higher in PLWH than in the general population. In addition, the impact of smoking seems also to differ in PLWH, with an increased risk of MI among currently smoking PLWH of 3-fold compared with controls, for a similar tobacco consumption (7). Other HIV-related factors have been reported to be involved, including immune activation despite ART, classes of ART regimen, coinfections with cytomegalovirus (CMV), gut microbial translocation, and HIV residual viremia (8–12).

Altogether, there is a need to uncover distinctive mechanisms underlying atherosclerosis development in PLWH and to identify optimal biomarkers that could improve the CVD risk stratification in PLWH and preventive strategies.

Growth differentiation factor-15 (GDF-15), a member of the transforming growth factor β family, is a stress-response cytokine that emerged as a novel biomarker for multiple diseases (13). While weakly expressed and involved in the metabolism under physiological conditions (14), its expression is markedly increased upon oxidative stress, aging, and inflammation (15–17). Regarding CVD, levels of GDF-15 have been associated with cardiovascular events in the general population with a history of acute coronary syndrome or stable coronary artery diseases (CAD) and also in absence of any CVD history (18–21). In patients with coronary atherosclerosis, GDF-15 levels have also been associated with chronic heart failure severity (22). However, GDF-15 levels and their association with diseases in PLWH remain poorly studied. Recent studies report increased GDF-15 circulating levels in ART-treated PLWH compared to controls, although the underlying mechanisms of such increase remain unknown (23, 24).

Coronary computed tomography angiography (CCTA) allows the most optimal non-invasive characterization and quantification of coronary atherosclerosis. Coronary plaque burden in general is considered a specific added value for cardiovascular risk stratification. More specifically, non-calcified lipid-rich necrotic core plaques are more prone to rupture in comparison to calcified plaques. Several coronary plaque high-risk features have been described on CCTA that help identify patients at risk of future coronary events (25). Among those characteristics, low-attenuation plaque (LAP) has been recently reported as a strong predictor of myocardial infarction in a patient presenting with stable chest pain (26). In PLWH, most studies revealed increased prevalence and size of atherosclerotic plaques. Our group recently reported a 2- to 3-fold higher prevalence and volume of non-calcified coronary plaque in asymptomatic PLWH compared to HIV-uninfected healthy volunteers in a prospective study (12).

Herein, we aimed to evaluate the association between different potential biomarkers, including GDF-15, and subclinical atherosclerotic lesions both in PLWH and uninfected controls. To this extent, we retrospectively measured GDF-15 in the plasma of participants with or without HIV infection who underwent CCTA. In addition, other related biomarkers linked with immunometabolism were tested: fibroblast growth factor 21 (FGF-21), a cytokine associated with insulin resistance and metabolism in PLWH (27), soluble urokinase plasminogen activator receptor (suPAR), a marker for monocyte, T-cell, and plasminogen activation associated with non-AIDS events in PLWH (28, 29), and GLP-2, a marker of microbial translocation with a role in tissue protection and improvement of metabolic function (30, 31). Moreover, high-sensitivity C-reactive protein (hsCP) as a representative of inflammation and IgG levels against common herpesviruses (CMV, EBV) were also measured.

## Methods

### Study design and population

This cross-sectional study was nested in the prospective multicentric Canadian HIV and Aging Cohort Study (CHACS), in which study design and protocol have been previously reported (32). In brief, PLWH over the age of 40 or having lived with HIV for at least 15 years have been recruited in HIV clinics between 2012 and 2018, with the majority being men who have sex with men. HIV-uninfected controls aged 40 years or older were recruited at the same medical centers. CHACS participants without symptoms or history of CAD and with a 10-year Framingham risk score ranging from 5 to 20% (classified as low to intermediate cardiovascular risk) were invited to participate in the cardiovascular imaging substudy. Data on CAD risk factors, demographics, and HIV disease were collected prospectively.

### Coronary CT

A 256-slice CT scanner (Brilliance iCT, Philips Healthcare, the Netherlands) was used to perform both non-contrast cardiac CT and CCTA in all participants. Before CCTA, patients received 50–75 mg of metoprolol orally if their heart rate was >60 beats per minute and 0.4 mg of nitroglycerin sublingually, in absence of contraindications. For CCTA, the contrast agent was injected at 5 ml/s, using 370 mg/ml iopamidol. A hybrid iterative reconstruction algorithm was used for image reconstruction (Philips iDose, Philips Healthcare). Plaque volumetric analysis was performed in multiplanar reformat (MPR). Coronary plaque analysis was performed using CCTA images as previously described (12, 33). Plaque volume has been assessed by using a semiautomated software (Aquarius iNtuition 4.4.6; TeraRecon) and analysis was performed in multiplanar reformat. Proximal and distal plaque boundaries were established visually, and segmentation was made by using the software with manual adjustment when plaque delimitation was inaccurate. Plaque composition was assessed using attenuation-stratified measurements in the plaque volume. Total plaque volume (TPV) per participant was defined as the sum of all attenuation-stratified measurements, whereas total low-attenuation plaque volume (LAPV) was defined as the sum of all plaque components with ≤30 HU. Imaging studies were performed at the Centre Hospitalier de l'Université de Montréal (CHUM), Canada, and interpreted by a board-certified cardiothoracic radiologist, blinded to HIV status.

### Laboratory measurements

Plasma HIV-1 p24 antigen/antibody and confirmation by Western blot were used to establish the diagnosis of HIV infection by clinical laboratories of the participating sites. Plasma HIV viral load was quantified with the Abbott Real-Time HIV-1 assay (Abbott Laboratories, Chicago, Illinois). Plasma samples of study participants have been collected in EDTA tubes and were stored at −80°C. Whole blood CD4 and CD8 T-cell counts were measured using 4-color flow cytometry by clinical laboratories of the participating sites.

### Measurement of plasma biomarkers

Levels of GDF-15, FGF-21, GLP-2, and suPAR were measured by enzyme-linked immunosorbent assay (ELISA) using commercialized kits (R&D Systems, Minneapolis, MN, USA and Merck Inc., MA, USA for GLP-2). Levels of anti-CMV IgG and anti-EBV were measured using the CMV IgG enzyme immunoassay test kit (GenWay Biotech, San Diego, California) and EBV IgG enzyme-linked immunosorbent assay (ELISA; GenWay Biotech, San Diego, California), respectively. All measures were performed in duplicate as per manufacturers' instructions.

### Statistical analyses

For all variables, medians with interquartile range were calculated. Unpaired comparisons were conducted using *T*-tests or Mann–Whitney *U*-tests, as appropriate. The Spearman rank correlation test was used to identify associations between 2 quantitative measures. *P*-values of <0.05 were considered significant. To control for potential confounding by classic cardiovascular risk factors on the association between tested biomarkers and coronary atherosclerosis, multivariable logistic regression analyses were performed. Total plaque volume and LAPV were dichotomized as absent or present. A parsimonious method was used to build the multivariable model. Age, sex, and smoking were kept in the model *a priori*. Classical risk factors (age, sex, smoking, diabetes, hypertension, and BMI > 30) were entered into the model sequentially and kept into the model if they modified the association between the tested biomarker and TVP by more than 10%. Data were analyzed with STATA 16.0 (StataCorp, College Station, TX, USA) and Graphpad Prism 8.0 (GraphPad Software, Inc., San Diego, CA, USA).

### Patient consent statement

The study was approved by the CHUM and all the participating centers. All participants provided written informed consent. The study was conducted in accordance with the principles of the Declaration of Helsinki.

## Results

### Study participant characteristics

A total of 147 participants from the cardiovascular imaging sub-study were included, including 95 ART-treated HIV^+^ participants and 52 uninfected controls. The median ages of HIV^+^ participants and controls were 56 (interquartile range [IQR], 52–61) and 55 (IQR 49–62) years, respectively, and 86% of participants were males (Table 1). Compared to controls, PLWH presented a significantly higher frequency of diabetes (9 vs. 0%, *p* = 0.03), hypertension (35 vs. 23%, *p* = 0.008), statin use (35 vs. 10%, *p* = 0.03), and smoking history (current smoking 25 vs. 10%, *p* = 0.03). Conversely, PLWH exhibited significantly lower levels of LDL (2.51 vs. 3.25 mmol/l, *p* < 0.001) and total cholesterol (4.66 vs. 5.26 mmol/l, *p* < 0.001) and had a lower body mass index than controls (25 vs. 27, *p* = 0.004). PLWH had a median CD4 and CD8 T-cell counts of 594 and 710 cell/ul, respectively, and a median CD4/CD8 T-cell ratio of 0.86. Of note, the controls CD4 and CD8 T-cell counts have not been measured. Regarding ART, 44% were receiving integrase inhibitor-based regimens and 35% protease inhibitors-based regimens.

**Table 1:** Clinical characteristics of study participants (*n* = 147).

**Characteristics**	**PLWH (*n* = 95)**	**Controls (*n* = 52)**	***p*-value**
**Age, years**			0.39
Median (IQR)	56 (52–61)	55 (49–62)	
Range	44–74	39–75	
**Sex, no. (%)**			**0.03**
Male	86 (90%)	40 (77%)	
Female	9 (10%)	12 (23%)	
Diabetes	9 (9%)	0 (0%)	**0.03**
Hypertension	33 (35%)	12 (23%)	**0.008**
Statin Use	33 (35%)	5 (10%)	**0.03**
**LDL, mmol/l**			**<0.001**
Median (IQR)	2.51 (2.02–3.38)	3.25 (2.70–3.93)	
Range	1.05–6.04	2.18–5.45	
**Total cholesterol**			**<0.001**
Median (IQR)	4.66 (3.95–5.40)	5.26 (4.90–6.08)	
Range	2.70–8.52	3.75–8.17	
**Body mass index, kg/m** ^**2**^			**0.004**
Median (IQR)	25.0 (21.5–27.5)	27.0 (24.0–31.0)	
Range	14.0–37.0	19.0–44.0	
Waist circumference, cm	92.5 (85.0–102.8)	95.0 (86.5–104.0)	0.59
**Smoking status**			**0.03**
Current smoker	24 (25%)	5 (10%)	
Former smoker	43 (45%)	19 (36%)	
Never smoked	27 (28%)	28 (54%)	
Presence of coronary plaque	66 (71%)	24 (51%)	**0.04**
**Total plaque volume**			**0.04**
Median (IQR)	92.0 (0–383)	16.9 (0–204)	
Range	0–1,725	0–1,148	
**Low-attenuation plaque volume**			**0.04**
Median (IQR)	17.4 (0–116)	1.0 (0–48)	
Range	0–694	0–427	
**CD4 T-cells/μL**			
Median (IQR)	594 (446–749)	N/A	
Range	117–1,723	N/A	
**CD8 T-cells/μL**			
Median (IQR)	710 (511–1,023)	N/A	
Range	145–2011	N/A	
**CD4:CD8**			
Median (IQR)	0.860 (0.546–1.140)	N/A	
Range	0.197–2.300	N/A	
HIV viral load, log10 copies/mL	<1.7	N/A	

PLWH, people living with human immunodeficiency virus; IQR, interquartile range; N/A, not applicable or not available. The bold values indicate the significant values of *p* < 0.05.

### Association between levels of biomarkers and presence of coronary plaque

In total, 66 (71%) and 24 (51%) of PLWH and controls (*p* = 0.04), respectively, presented coronary plaque (defined as TPV above 0 mm^3^). Notably, none of the participants presented total plaque without any low-attenuation plaque or the opposite. Both TPV and LAPV were significantly higher in PLWH compared to controls (*p* = 0.04 for both). In PLWH, the presence of coronary plaque, both TPV and LAPV, was associated with higher levels of GDF-15 (1,037 vs. 764 pg/ml, *p* = 0.04, Table 2), as well as higher levels of suPAR (2,772 vs. 2,062 pg/ml, *p* = 0.003). In uninfected controls, levels of GDF-15 were also increased in presence of coronary plaque (640 vs. 415 pg/ml, *p* < 0.001), as well as the level of anti-EBV IgG (42 vs. 30 Genway U, *p* = 0.005). Regarding other tested biomarkers, FGF-21, GLP-2, hsCRP, and anti-CMV IgG levels were not significantly different in participants with coronary plaque compared to those without. Notably, the levels of GDF-15 were increased in PLWH compared to controls, both for participants with (1,037 vs. 640 pg/ml, *p* < 0.001) or with the out presence of coronary plaque (811 vs. 415 pg/ml, *p* < 0.001, Supplementary Figure 1).

**Table 2:** Plasmatic levels of tested biomarkers in people living with HIV and controls depend on the absence or presence of coronary plaque.

**Biomarker**	**PLWH (*****n*** = **95)**		***p*-value**	**Controls (*****n*** = **52)**		***p*-value**
	**Absence of plaque**	**Presence of plaque**		**Absence of plaque**	**Presence of plaque**	
GDF-15 (pg/mL)	764 (716–1,760)	1,037 (584–1,112)	**0.04**	415 (290–413)	640 (419–973)	**<0.001**
FGF-21 (pg/mL)	172 (86–290)	224 (110–493)	0.44	189 (90–326)	184 (82–312)	0.73
GLP-2 (pg/mL)	1.9 (1.1–3.0)	1.8 (1.5–3.6)	0.84	2.8 (1.8–3.3)	2.2 (1.7–3.0)	0.60
suPAR (pg/mL)	2,062 (1,699–2,828)	2,772 (2,286–3,587)	**0.003**	1,864 (1,660–2,225)	2,331 (1,790–3,157)	0.08
hsCRP (mg/L)	5.0 (5.0–5.0)	5.0 (5.0–5.0)	0.14	5.0 (5.0–5.8)	5.0 (5.0–5.0)	0.54
Anti-CMV IgG (IU/mL)	27.2 (23.5–32.2)	28.7 (23.7–31.4)	0.62	4.9 (0.0–24.2)	13.6 (0.0–22.9)	0.68
Anti-EBV IgG (Genway U)	44.2 (35.9–47.3)	42.8 (30.2–47.8)	0.61	29.7 (16.8–42.6)	42.1 (33.7–48.0)	**0.008**

Medians and interquartile ranges are described. PLWH, people living with human immunodeficiency virus. The bold values indicate the significant values of *p* < 0.05.

### Correlation between levels of biomarkers and volumes of total plaque and low-attenuation plaque

**Figure 1:**
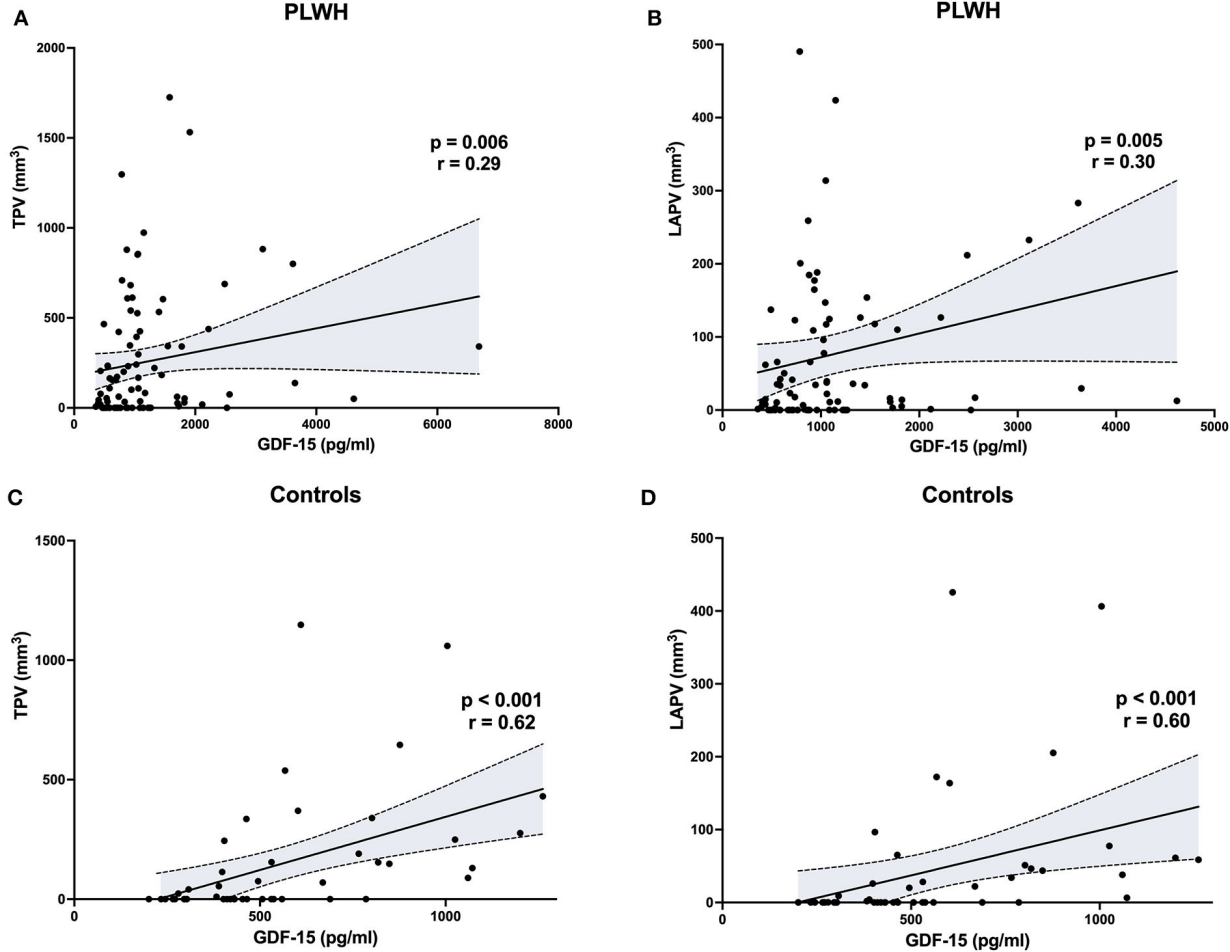
Correlations between total or low-attenuation plaque volume (TPV, LAPV) and GDF-15 levels in people living with HIV and controls. The Spearman rank correlation test was used to identify associations. PLWH, people living with human immunodeficiency virus.

The GDF-15 levels were significantly correlated with both TPV and LAPV in PLWH (*r* = 0.29, *p* = 0.006 and *r* = 0.30, *p* = 0.005, respectively, Figure 1 and Table 3), as well as in controls (*r* = 0.62, *p* < 0.001 and *r* = 0.60, *p* < 0.001, respectively). The suPAR levels were correlated with TPV and LAPV only in PLWH (*r* = 0.29, *p* = 0.01 for both). Regarding other biomarkers, TPV and LAPV were significantly associated with levels of hsCRP in PLWH only (*r* = 0.26, *p* = 0.03 and *r* = 0.28, *p* = 0.02, respectively), and tended to be correlated with anti-EBV IgG levels in controls (*r* = 0.27, *p* = 0.07, and *r* = 0.26, *p* = 0.08, respectively). All other tested biomarkers were not correlated with plaque volume.

**Table 3:** Correlations between clinical measures and total plaque volume or low-attenuation plaque volume in people living with HIV and uninfected controls.

**Biomarker**	**Correlation with total plaque volume**			
	**PLWH (*****n*** = **95)**		**Controls (*****n*** = **52)**	
	***r***	***p*-value**	***r***	***p-*value**
GDF-15 (pg/mL)	0.29	**0.006**	0.62	**<0.001**
suPAR (pg/mL)	0.29	**0.01**	0.34	0.07
FGF-21 (pg/mL)	0.23	0.05	−0.06	0.72
GLP-2 (pg/mL)	0.07	0.66	0.12	0.58
hsCRP (mg/L)	0.26	**0.03**	−0.15	0.54
Anti-CMV IgG (IU/mL)	0.11	0.31	−0.02	0.89
Anti-EBV IgG (Genway U)	−0.13	0.24	0.27	0.07
**Biomarker**	**Correlation with low-attenuation plaque volume**			
	**PLWH (*****n*** = **95)**		**Controls (*****n*** = **52)**	
	***r***	***p*** **-value**	***r***	***p*** **-value**
GDF-15 (pg/mL)	0.30	**0.005**	0.60	**<0.001**
suPAR (pg/mL)	0.29	**0.01**	0.34	0.06
FGF-21 (pg/mL)	0.22	0.06	−0.02	0.91
GLP-2 (pg/mL)	0.10	0.53	0.14	0.52
hsCRP (mg/L)	0.28	**0.02**	−0.15	0.54
Anti-CMV IgG (IU/mL)	0.08	0.44	−0.02	0.88
Anti-EBV IgG (Genway U)	−0.13	0.24	0.26	0.08

PLWH, people living with human immunodeficiency virus. The bold values indicate the significant values of *p* < 0.05.

### Multivariate analysis of the association between biomarkers levels and coronary plaque volume

The influence of classical cardiovascular risk factors (age, sex, pack-years of smoking, hypertension, diabetes, family history of premature cardiovascular disease, body mass index, and use of statins) on the association between biomarkers and coronary plaque was assessed in a multivariate model (Table 4). In controls, GDF-15 levels were found to be independently associated with LAPV, with each increase of 1 standard deviation (SD) associated with an increased odds ratio (OR) for coronary atherosclerosis of 35.08 (95% CI = 1.18->999, *p* = 0.04) after adjustment. In PLWH, whereas GDF-15 levels were associated with LAPV in the crude analysis (OR 2.27, *p* = 0.05), the association was lost after adjustment due to confounding by smoking (Supplementary Table 1). In PLWH, suPAR levels were also associated with LAPV in the crude analysis (OR 1.90, 95% CI = 1.03–3.52, *p* = 0.04), but not in the multivariate model (Table 4). In controls, anti-EBV IgG levels were found to be associated with LAPV, independently of classical cardiovascular risk factors (OR 3.51, 95% CI = 1.17–10.50, *p* = 0.02, Table 4).

**Table 4:** Crude and adjusted association between biomarkers of interest and presence of subclinical low-attenuation coronary atherosclerosis (LAPV>0) in participants with and without HIV infection.

	**PLWH (*****n*** = **95)**				**Controls (*****n*** = **52)**			
	**Crude OR [95% CI]**	***p*-value**	**Adjusted OR [95% CI]**	***p*-value**	**Crude OR [95% CI]**	***p*-value**	**Adjusted OR [95% CI]**	***p*-value**
GDF-15 (pg/mL)	**2.27** (0.99–5.20)	**0.05**	1.37 (0.65–2.90)	0.67	**139.71** (5.60->999)	**0.003**	**35.08** (1.18–>999)	**0.04**
suPAR (pg/mL)	**1.90** (1.03–3.52)	**0.04**	1.42 (0.75–2.69)	0.28	3.61 (0.88–14.79)	0.07	5.81 (0.81–41.89)	0.08
FGF-21 (pg/mL)	0.92 (0.62–1.37)	0.69	0.81 (0.51–1.30)	0.38	0.72 (0.16–3.19)	0.66	0.58 (0.06–5.55)	0.64
GLP-2 (pg/mL)	1.00 (0.60–1.69)	0.99	0.64 (0.29–1.40)	0.26	1.37 (0.33–5.74)	0.67	4.71 (0.47–46.80)	0.19
hsCRP (mg/L)	1.46 (0.54–3.96)	0.46	1.25 (0.59–2.62)	0.56	0.77(0.40–1.48)	0.42	0.77 (0.40–1.48)	0.42
Anti-CMV IgG (IU/mL)	1.09 (0.57–2.07)	0.79	0.91 (0.46–1.78)	0.78	1.06 (0.57–1.98)	0.85	1.16 (0.61–2.22)	0.65
Anti-EBV IgG (Genway U)	0.84 (0.49–1.43)	0.51	0.98 (0.56–1.71)	0.95	**2.53** (1.26–5.09)	**0.009**	**3.51** (1.17–10.50)	**0.02**

Multivariable models are adjusted for age, sex, pack years of smoking, hypertension, diabetes, family history of premature cardiovascular disease, body mass index, and use of statins. Model building strategy is parsimonious; potential confounders are entered into the model sequentially and kept into the model if they modify the point estimate for the OR by ≥10%. OR, odds ratio; LAPV, low-attenuation plaque volume; PLWH, people living with human immunodeficiency virus. The bold values indicate the significant values of p < 0.05.

### Association between GDF-15 levels, smoking, and coronary plaque volume

**Figure 2:**
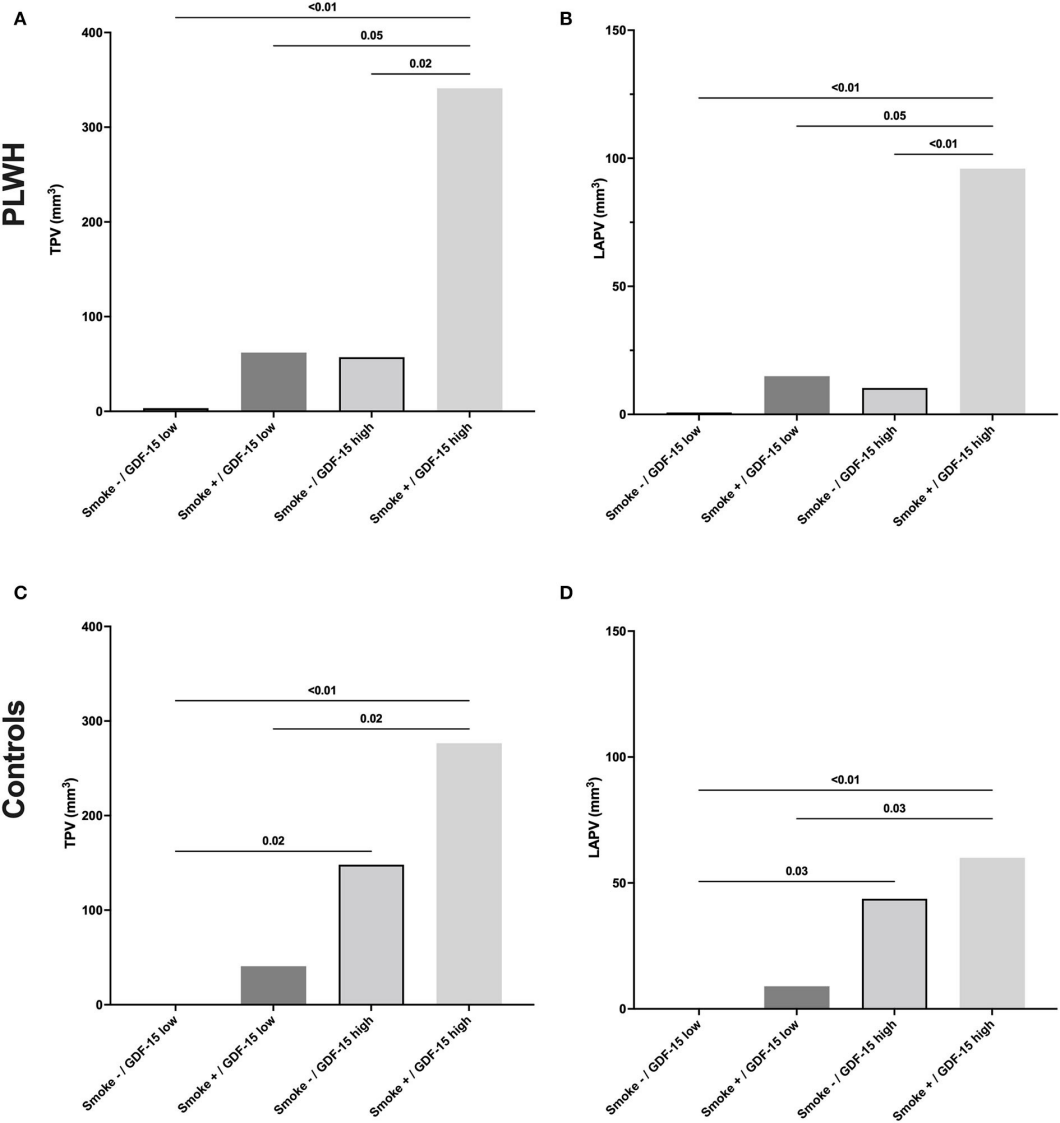
Total or low-attenuation plaque volume depending on the smoking status and GDF-15 level in PLWH and controls. GDF-15 levels below the median value of our participants (766 pg/ml) were defined as “low,” whereas levels above this value were defined as “high.” PLWH, people living with human immunodeficiency virus; TPV, total plaque volume; LAPV, low-attenuation plaque volume.

To further investigate the combined influence of GDF-15 and smoking on coronary plaque volume, we assessed plaque volume in four distinct groups of participants (Figure 2), depending on smoking status (<5 pack-years vs. >5 pack-year) and GDF-15 levels (GDF-15 below vs. higher than the median level, 766 pg/ml). For both PLWH and controls, participants without smoking and with low GDF-15 levels had no detectable coronary plaque. In both PLWH and controls, participants with high GDF-15 levels and a history of smoking (>5 pack-year) had significantly higher plaque volume (TPV and LAPV) than participants with low GDF-15 levels, with or without a smoking history. In controls, non-smoker participants with high levels of GDF-15 had significantly higher plaque volume than those with low GDF-15 levels (*p* = 0.02). In contrast to PLWH, only the association of high GDF-15 levels and smoking was associated with a significantly higher plaque volume in controls, whereas smoking or increased GDF-15 levels only were not.

## Discussion

In this cross-sectional study, we analyzed the association between different biomarkers and coronary plaque volume in 95 PLWH and 52 HIV-uninfected controls who all underwent CCTA. Previous data have shown that non-AIDS comorbidities in PLWH are known to be influenced by metabolic, inflammatory, and viral factors (34–36); thus we tested markers related to such aspects, including GDF-15, suPAR, FGF-21, GLP-2, CRP, anti-CMV IgGs, and anti-EBV IgGs.

In this study, we found that in both PLWH and uninfected controls, elevation in GDF-15 levels was significantly associated with TPV and LAPV, which constitutes the high-risk portion of atherosclerotic plaques. Whereas, GDF-15 levels have already been reported to be associated with CVD in the general population, this is the first report of the association between GDF-15 levels and subclinical CVD in PLWH. Among both PLWH and controls, the level of GDF-15 correlated with both TPV and LAPV, and higher levels were associated with a higher prevalence of plaque. In line with recent studies from 2022, one by Domingo and collaborators and the other by Agarwal and collaborators (23, 24), levels of GDF-15 were found to be increased in PLWH compared to controls. Regarding the absolute levels, median GDF-15 levels were comparable to our study in the study of Domingo et al. (median 724 pg/ml), whereas those levels were higher in the study from Agarwal et al. (median 1507 pg/ml for PLWH and 671 pg/ml for controls). However, the absolute levels of GDF-15 should be compared with caution, in light of the known influence of age and ethnicity on GDF-15 levels (13, 24).

Interestingly, we also found that the association between GDF-15 and a coronary plaque was independent of classical CV risk factors only in controls but confounded by tobacco smoking in PLWH. In HIV observational studies, tobacco smoking is known to be a frequent confounding factor when assessing CVD risk (37), and the high rate of tobacco smoking constitutes a well-known factor of increased CVD risk in PLWH. However, the attributable risk of tobacco smoking to CVD seems to be also disproportionally increased in this population (7). The functions of GDF-15 remain poorly understood, with opposing roles on inflammatory pathways in a context-dependent manner and smoking might increase GDF-15 levels, as reported by Wada et al. in HIV-uninfected controls (38). Whether smokers had also increased subclinical CVD was, however, not assessed in this latter study, and the mechanism underlying this association remains unknown. Altogether, whether GDF-15 constitutes a causative risk factor or a CVD biomarker remains to be defined, although mounting evidence suggests a direct role for GDF-15 in accelerating atherosclerosis (39, 40). Although out of the scope of this study, our results are the first to propose a synergistic effect of GDF-15 and tobacco smoking on coronary plaque formation exclusively in PLWH and not in the general population. This distinctive effect of smoking in PLWH needs to be further explored, as it could be linked with the increased risk of CVD in PLWH compared to the general population.

Regarding other tested biomarkers, we also found an association between suPAR levels and the presence of coronary plaque only in PLWH. Although this association was lost after adjustment in the multivariate model, this marker of non-AIDS (including cardiovascular) comorbidities might be of interest for further studies. In controls, an unexpected association between coronary plaque and anti-EBV IgG levels was found as well, which was independent of confounding factors in the multivariate model. Although the role of EBV as a trigger for inflammatory or autoimmune pathologies is well-known for other diseases, such as recently for multiple sclerosis (41), such an influence on atherosclerosis is still debated (42). This association needs thus to be further confirmed in larger and dedicated studies. Conversely, anti-CMV IgG levels were surprisingly not correlated with plaque volume in both groups, although CMV chronic infection has been reported to increase CVD risk in some studies (43, 44).

In addition to its cross-sectional nature, our study had several limitations. First, although recruitment was designed to minimize inter-group differences, included PLWH were more frequently males and presented higher rates of diabetes, hypertension, smoking, and statin use. However, those differences were taken into account in the multivariate models and might, in part, reflect the close monitoring and prompt preventive strategies in PLWH, indicated by the decreased levels of cholesterol and LDL. Second, ART regimens were not taken into account in the analysis, although most of the participants were not receiving protease inhibitors. Third, whether GDF-15 participates in the pathogenesis of high-risk coronary plaques remains to be better defined, as this biomarker was similarly correlated with TPV and LAPV both in PLWH and controls. Finally, the limited number of participants and their modest rates of coronary plaque resulted in insufficient power to analyze subgroups' characteristics or influences on cardiovascular events. However, this study could pave the way for larger prospective studies that will confirm the association between GDF-15 and subclinical atherosclerotic lesions.

In conclusion, we report herein that increased GDF-15 levels were associated with the presence of coronary artery plaques in people without HIV, independent of smoking, and other CV risk factors, including smoking. GDF-15 might be useful as a biomarker for CVD in the general population, although further studies will be needed to clearly define its additive prognostic impact and a clear cut-off will need to be set. Conversely, in PLWH, GDF-15, and smoking seemed to have a synergistic effect on coronary plaque volume. This distinctive effect of smoking in PLWH compared to uninfected controls opens new research avenues for the understanding of the increased risk of CVD in PLWH. Smoking cessation should remain a priority in the management of CVD in PLWH, and more research on the link between GDF-15 and plaque formation is warranted.

## Summary

In people living with HIV, GDF-15, and smoking have a synergistic effect on coronary plaque volume. Conversely, in people without HIV, increased GDF-15 levels are strongly associated with the presence of coronary artery plaques independently of cardiovascular risk factors.

## Data availability statement

The raw data supporting the conclusions of this article will be made available by the authors, without undue reservation.

## Ethics statement

The studies involving human participants were reviewed and approved by CHUM, Montréal, QC, Canada. The patients/participants provided their written informed consent to participate in this study.

## Author contributions

LR designed the study, prepared the first draft of the manuscript, and revised the final draft of the manuscript. LR, SI, NP, LS, and CB performed the experiments and analyzed the data. J-PR and MD supervised the work. All authors revised the initial draft, contributed to the article, and approved the submitted version.

## Funding

This study was funded by the Canadian Institute of Health Research (CIHR) Canadian HIV and Aging Cohort Study (Group Grant No. 284512); it is now supported by CIHR Grant 364423, CIHR Group Grant No. 398643, and Project Grant No. 399544; the National Institutes of Health (R01AG054324). The Canadian HIV and Aging Cohort Study was supported by the Clinical Care Management Core of the CIHR Canadian HIV Trials Network (CTN 272). This study was also financed by the Fonds de la Recherche Québec-Santé (FRQ-S): Réseau SIDA/Maladies Infectieuses and Thérapie Cellulaire; Réseau de Bioimagerie du Québec (FRQ-S); the CIHR (Grant Nos. MOP 103230 and PTJ 166049); the Vaccines and Immunotherapies Core of the CIHR Canadian HIV Trials Network (Grant No. CTN 257); Département de Radiologie, Radio-Oncologie et Médecine Nucléaire, University of Montreal; the National Institutes of Health; the Canadian Foundation for AIDS Research (Grant No. 02-512); and the CIHR-funded Canadian HIV Cure Enterprise (Team Grant No. HB2-164064). LR is a postdoctoral fellow supported by the Swiss National Science Foundation and the CIHR-CTN. SI is a postdoctoral fellow supported by the FRQ-S and CIHR-CTN. CT holds the Pfizer/Université de Montreal Chair on HIV translational research. MD receives a clinician-researcher salary award from FRQ-S. J-PR holds the Louis Lowenstein Chair in Hematology and Oncology, McGill University.

## Conflict of interest

The authors declare that the research was conducted in the absence of any commercial or financial relationships that could be construed as a potential conflict of interest.

## Publisher's note

All claims expressed in this article are solely those of the authors and do not necessarily represent those of their affiliated organizations, or those of the publisher, the editors and the reviewers. Any product that may be evaluated in this article, or claim that may be made by its manufacturer, is not guaranteed or endorsed by the publisher.

## Supplementary material

Supplementary Table 1 The parsimonious method was used to build the multivariable model. Age, sex, and smoking were kept in the model *a priori*. Classical risk factors (age, sex, smoking, diabetes, hypertension, and BMI > 30) were entered sequentially and kept into the model if they modified the association between the tested biomarker and TVP by more than 10%.

Supplementary Figure 1 Levels of circulating GDF-15 depending on the HIV status in participants with or without the presence of low-attenuation plaque volume. PLWH, people living with human immunodeficiency virus; LAPV, low-attenuation plaque volume.
