# Supplementary material for: Growth differentiation factor-15 as a biomarker of atherosclerotic coronary plaque: Value in people living with and without HIV
Source: Front Cardiovasc Med. 2022 Aug 26;9:964650. doi: 10.3389/fcvm.2022.964650 (PMC9458883; doi:10.3389/fcvm.2022.964650)
Supplement: Supplementary Table 1 — The parsimonious method was used to build the multivariable model. Age, sex, and smoking were kept in the model a priori. Classical risk factors (age, sex, smoking, diabetes, hypertension, and BMI > 30) were entered sequentially and kept into the model if they modified the association between the tested biomarker and TVP by more than 10%. [file Table_1.docx]

| **Model** | **OR** | ***p*-value** | **Final model** |
| --- | --- | --- | --- |
| GDF-15 | GDF15=2.27 (0.99-5.20) | 0.05 |  |
| GDF-15+age | GDF15=1.88 (0.84-4.22)  Age=1.07 (0.98-1.16) | 0.13  0.12 | yes |
| GDF-15+age+sex | GDF15=1.86 (0.77-4.47)  Age=1.07 (0.98-1.16)  Sex 1 vs 2 =0.94 (0.08-10.68) | 0.17  0.12  0.96 | no |
| GDF-15+age+ Pack_years | GDF15=1.54 (0.71-3.37)  Age=1.07 (0.98-1.16)  **Pack_years= 1.04 (1.01-1.07)** | 0.27  0.12  **0.01** | yes |
| GDF-15+age+ Pack_years+Hypertension | GDF15=1.55 (0.71-3.38)  Age=1.07 (0.98-1.16)  Pack_years=1.04 (1.01-1.07)  Hypertension 1 vs 2=1.13 (0.36- 3.52) | 0.27  0.16  0.01  0.83 | no |
| GDF-15+age+ Pack_years+ diabetes | GDF15=1.17 (0.53-2.58)  Age =1.07 (0.98-1.16)  Pack_years =1.04 (1.01-1.07)  Diabetes 1 vs 2 =>999.99 ( <0.001- >999.99) | 0.69  0.12  0.02  0.96 | no |
| GDF-15+age+ Pack_years+ bmi | GDF15 =1.47 (0.68-3.15)  Age=1.07 (0.98-1.17)  Pack_years=1.04 (1.01-1.07)  BMI= 0.93 (0.83-1.06) | 0.33  0.12  0.01  0.27 | no |
| GDF-15+age+ Pack_years+ statins | GDF15=1.37 (0.65-2.90)  Age=1.06 (0.98-1.16)  Pack_years=1.04 (1.01-1.07)  Statins 0 vs 1 0.32 (0.09-1.13) | 0.41  0.17  0.02  0.08 | Final model |

**Supplementary Table 1**. Detailed model building. Model building strategy is parsimonious; potential confounders are entered into the model sequentially, and kept into the model if they modify the point estimate for the OR by >= 10%. OR: Odds ratio.

volume.
