# Supplementary figures and images for: Growth differentiation factor-15 as a biomarker of atherosclerotic coronary plaque: Value in people living with and without HIV
Source: Front Cardiovasc Med. 2022 Aug 26;9:964650. doi: 10.3389/fcvm.2022.964650 (PMC9458883; doi:10.3389/fcvm.2022.964650)

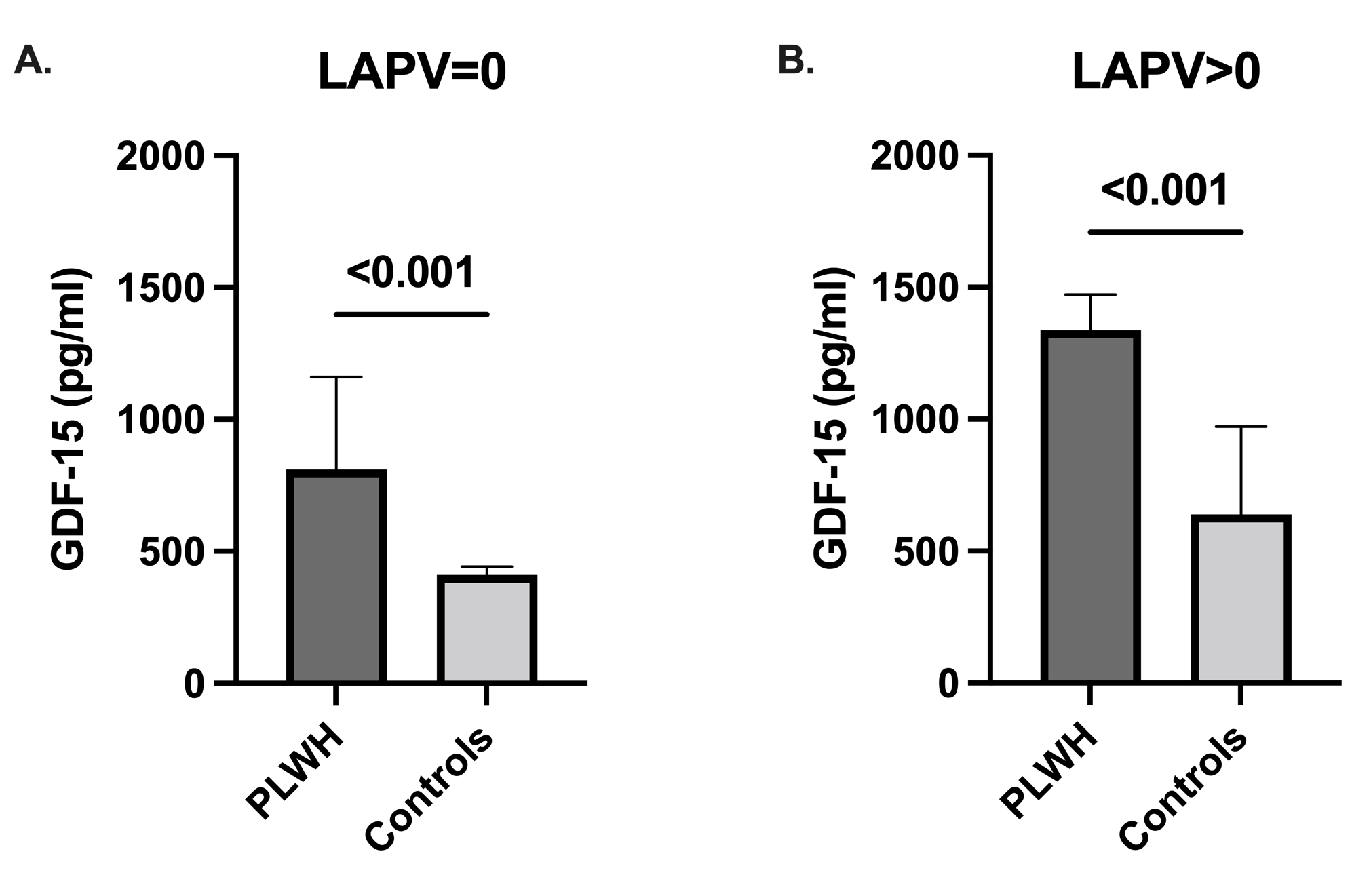

Supplement: Supplementary Figure 1 — Levels of circulating GDF-15 depending on the HIV status in participants with or without the presence of low-attenuation plaque volume. PLWH, people living with human immunodeficiency virus; LAPV, low-attenuation plaque volume. [file Image_1.TIFF]
